# Genome-wide association study and gene network analysis of drought tolerance in wheat during early growth

**DOI:** 10.3389/fpls.2026.1775894

**Published:** 2026-03-30

**Authors:** Amira M. I. Mourad, Ahmed Sallam, Shamseldeen Eltaher, Andreas Börner, Yasser S. Moursi

**Affiliations:** 1 Genebank Department, Leibniz Institute of Plant Genetics and Crop Plant Research (IPK), Gatersleben, Germany; 2 Department of Agronomy, Faculty of Agriculture, Assiut University, Assiut, Egypt; 3 Faculty of Biotechnology, Badr University in Assiut (BUA), Assiut, Egypt; 4 Department of Genetics, Faculty of Agriculture, Assiut University, Assiut, Egypt; 5 Department of Plant Biotechnology, Faculty of Biotechnology, University of Sadat City (USC), Sadat City, Egypt; 6 Department of Botany, Faculty of Science, Fayoum University, Fayoum, Egypt

**Keywords:** drought, gene network, germination, GWAS, seedling, *Triticum aestivum* L.

## Abstract

**Introduction:**

Drought is one of the most damaging abiotic stresses, reducing seed germination, impairing seedling establishment, and ultimately decreasing crop yield. The objectives of the present study were to identify associated SNP markers and genes associated with drought tolerance in wheat at early developmental stages and to explore gene networks that reveal gene–gene interactions under drought stress.

**Methods:**

A total of 168 genotypes were tested for drought tolerance under 20% PEG (drought stress) and 0% PEG (control). Genome-wide association study, annotation analyses, gene network analysis were performed.

**Results:**

High genetic variation was observed among genotypes for all traits scored under both conditions. The GWAS identified 130 and 128 significant SNPs under drought and control conditions, respectively. Gene annotation identified 98 genes responsive to drought stress, of which 54 have been previously reported to be associated with drought tolerance. Ten SNPs were common between control and drought treatments, nine of which were located within genes controlling variation in germination percentage. Gene network analysis of these nine gene models showed that they were organized into eight distinct pathways.

**Discussion:**

These networks were regulated by master genes encoding proteins with diverse biological functions, including rRNA and tRNA methyltransferases, protein kinases, and potassium transmembrane transporters. These findings provide fundamental insights into the genetic basis of drought tolerance in wheat. These results indicate that GWAS is a robust and instrumental approach to identifying the associated genes. The network analysis explored the interactions among several genes, not the effect of each gene individually, which will help us to understand the drought tolerance during early stages in wheat. These findings are fundamental to better understand drought tolerance in wheat.

## Introduction

Climate changes lead to extended periods with low water availability due to the growing demand for food, making breeding for drought tolerance a high priority. Drought hinders plant development, growth, and productivity. Among the various abiotic stresses, drought imposes the highest impact on crop yield (Chaves et al., 2002). In the UK, mild drought causes wheat yield loss, ranging from 30% to 60% (Dodd et al., 2011). Drought tolerance is a very complex trait controlled by many factors including drought severity, stress duration, plant growth stage, plant genotype, and patterns of gene expression (reviewed by Nezhadahmadi et al., 2013). The negative effects of drought on plants were demonstrated throughout the entire plant life cycle, including seed germination, seedling establishment, flowering, and grain filling (Oguz et al., 2022). Successful seed germination and seedling formation are among the most sensitive stages in plant growth and establishment, indicating their importance for plant growth under water-deficit conditions (Mickky and Aldesuquy, 2017; Wolny et al., 2018; Reddy et al., 2020).

Despite the success in improving some valuable traits, conventional breeding for abiotic stress tolerance is still far from achieving the same success and is more complicated. This complication is attributed to the polygenic control of these traits and to the crosstalk between various mechanisms to confer tolerance against abiotic stresses (i.e., many quantitative trait loci) (Fita et al., 2015; Sallam et al., 2026b). Therefore, applying molecular approaches, including identifying and mapping these quantitative trait loci and exploring the associated alleles/genes nearby or in the vicinity of such loci, is very helpful to elucidate and improve drought tolerance in crop plants. Several studies have identified several quantitative loci that contributed to the diverse genotypic response to drought tolerance. In wheat, several QTLs for drought tolerance during seed germination and seedling formation were identified (Hao et al., 2003; Landjeva et al., 2008; Zhang et al., 2013, 2014; Czyczyło-Mysza et al., 2014; Nagel et al., 2014; Ashraf et al., 2015; Dodig et al., 2015; Ma et al., 2017, 2025; Gahlaut et al., 2017; Iannucci et al., 2017; Zuo et al., 2019; Ren et al., 2021; Sallam et al., 2022, 2024a; Ramappa et al., 2023; Siddiqui et al., 2023; Hasanpour et al., 2023; Liu et al., 2025b; Frantová et al., 2025; Guo et al., 2025).

For breeding purposes, the identification of top-performing genotypes is helpful to make the best crosses, thereby saving time, money, and effort. The multi-trait selection approaches simplify this process by enabling the simultaneous selection of a set of desirable traits. The multi-trait genotype–ideotype distance index (MGIDI) was used to select the superior genotypes in several crop species (Debnath et al., 2024). Elite genotypes with superior performance under drought were identified using MGIDI in wild wheat (Pour-Aboughadareh and Poczai, 2021; Mohi-Ud-Din et al., 2025).

It has been demonstrated that large sets of genes work together as networks, tailoring the response of plants to drought. Drought stress imposes physiological, biochemical, cellular, and molecular changes, as some metabolites accumulate as a response to water deficit such as sugars, sugar alcohols, amines, and amino acids. These metabolites act as osmolytes, antioxidants, or scavengers that help plants withstand drought, and these metabolites operate coordinately as metabolic networks (Seki et al., 2007). These networks include signaling networks, metabolic networks, phosphorylation networks, gene expression regulatory networks, and transcriptional and posttranscriptional regulatory networks (Seki et al., 2007; Takahashi et al., 2018). The gene products of drought-inducible genes are classified into two main categories: functional proteins that are involved in drought tolerance and regulatory proteins that are involved in the regulation of transcription and signal transduction. The first group may include molecules such as chaperones, late embryogenesis abundant (LEA) proteins, osmotin, antifreeze proteins, mRNA-binding proteins, and osmolyte-synthesizing enzymes. The second group includes various transcription factors, protein phosphatases, protein kinases, enzymes involved in phospholipid metabolism, and other signaling molecules (Shinozaki and Yamaguchi-Shinozaki, 2007).

Identifying candidate genes with strong potential for breeding drought-tolerant crops remains a significant challenge (Krannich et al., 2015). Thus, deciphering the molecular mechanisms behind plant responses to drought stress is challenging because of the intricate interactions among a multitude of genes. As an example of this complexity, in wheat, a regulatory network analysis identified 7.2 million genome-wide interactions spanning 5,947 TFs and 127,439 candidate genes (Chen et al., 2023). In rice, using a network-based analysis, 2,607 genes revealed drastic changes under drought; among them, 1,392 genes were highly interlinked, forming 15 gene modules. One of these modules consisted of 134 genes associated with drought in both drought-tolerant and drought-sensitive genotypes (Zhang et al., 2012). In wheat, network-based analysis identified novel genes responsive to early drought (Barratt et al., 2023). Producing more knowledge about the complex networks and the crosstalk during drought will make more options available to improve drought tolerance in crop plants. The QTL analysis provides important information on the genes controlling drought tolerance. Investigating gene networks among these genes is therefore crucial for identifying hub regulators and new breeding targets (Schierenbeck et al., 2023), capturing the complexity beyond single genes (Nouraei et al., 2022), and translating to breeding and markers (Schierenbeck et al., 2023). Gene network analysis helps researchers to move from a long list of associated genes to mechanistic, interconnected pathways and key regulators, greatly improving the power of understanding and breeding for drought-tolerant wheat.

The objectives of the current study were to 1) detect the genetic factors controlling drought response in wheat at early stages and 2) identify the gene networks involved in drought response.

## Materials and methods

### Plant material

A total of 168 bread wheat (*Triticum aestivum* L.) genotypes originating from 22 countries were evaluated for drought tolerance at early stages. A detailed list of the genotypes is provided in **Supplementary Table 1**. The genotypes were collected from the USDA.

### Germination and seedling establishment assay

All genotypes were tested under drought stress using a randomized complete block design (RCBD) with three biological replicates. For each genotype, 20 seeds were rinsed with distilled water, surface-sterilized with 1% sodium hypochlorite (NaOCl) for 10 min, and subsequently rinsed several times with deionized water. Sterilized seeds were placed in Petri dishes lined with two layers of Whatman No. 1 filter paper and moistened with 10 mL of the corresponding solutions, either tap water for the control or 20% polyethylene glycol (PEG 6000) for drought stress. Thereafter, Petri dishes were incubated at 20°C in complete darkness. To maintain consistent drought levels, the treatment solutions were renewed every other day throughout the experiment.

Germination was scored every 24 h for 10 days, with a seed considered germinated when the radicle reached 2 mm in length. On day 10, several germination traits were recorded. Seedling traits, including shoot length (SL), root length (RL), and the number of roots (NoR), were assessed. Ten seeds per genotype were placed on moistened rolled paper towels following the method of Hetz et al. (1996). The rolls were vertically positioned in 1-L glass beakers containing either tap water or 20% PEG solution. Solutions were refreshed every 2 days. After 12 days, the experiment was terminated, and the traits were measured or calculated as follows:

**Trait measurement and calculations:**

Germination Percentage (G%):


$$\text{G}\%=\frac{\text{n}}{\text{N}}\text{x }100$$

Where n is the number of germinated seeds on the 10th day, and N is the total number of seeds sown (20).

Germination Pace (GP) was calculated according to the following equation


$$\text{GP}=\frac{\text{N}}{\sum (\text{n x g})}\text{x }100$$

Where N is the total number of germinated seeds, n is the number of newly germinated seeds on day g, and *g* represents each day of observation (1, 2, 3, …).

Root and Shoot were measured manually in centimeters using a ruler.

Root/Shoot Ratio (RSR): Calculated as the ratio of RL to SL on day 12.

Number of Roots (NoR): Total root number counted visually on day 12.

Drought Tolerance Index (STI):


$$\text{DTI}=\frac{\text{Trait value under drought}}{\text{Trait value under control}}\text{x }100$$

Trait abbreviations are followed by a suffix indicating the treatment condition: **C** for control and **D** for drought stress.

Mean values across replicates were used for further analysis, including genome-wide association studies (GWAS).

### Genetic analyses

#### DNA extraction and genotyping

DNA was extracted from seedling leaves (6-day old) from each genotype using the Thermo Scientific GeneJET Plant Genomic DNA Purification Mini Kit protocol. DNA concentration for each genotype was measured using a NanoDrop (Bio-Rad Laboratories, Hercules, CA, USA) equipment. DNA extraction was performed at the Department of Genebank, Leibniz Institute of Plant Genetics and Crop Plant Research, Germany.

The DNA samples were sent for genotyping by the GmbH Trait Genetics Section, Gatersleben, Germany, using a 25K SNP Illumina Infinium Array (25K) as described by Aleksandrov et al. (2021). The SNP array generated a total of 24,145 SNPs, which were reduced after filtration to 21,093 SNPs. The filtration criteria were performed according to Alqudah et al. (2020a). The population structure for this population was previously reported in Sallam et al. (2024b). The LD decay for each wheat subgenome, as well as across the entire genome, was previously reported by Mourad et al. (2020).

GWAS was performed between all SNP markers, and all traits were scored under normal and drought stress using the Visualization-enhanced and Parallel-accelerated (rMVP) package. GWAS models, including a general linear model (GLM), a mixed linear model (MLM), and a fixed and random model [circulating probability unification (FarmCPU)], were applied to identify significant SNP markers under both conditions. kinship (Kin), principal coordinate analysis (PCA), and PCA + Kin were included in each model to correct the effect of population structure, resulting in nine GWAS models. The fit GWAS model for each trait was selected based on the distribution of the expected and observed *p*-values in the quantile–quantile plot (Q-Q plot). The model was selected when most points (observed vs. expected −log10(p)) lie on the diagonal; only the extreme tail deviates upward (true associations) (Alqudah et al., 2020b). For each evaluated trait, markers were declared significantly associated with the target traits when *p* ≤ 0.001 (−log10 p ≥ 3.0). Using 0.001 as a working cutoff is consistent with the recommendations that overly strict Bonferroni thresholds can be unnecessarily conservative in such GWAS designs (Kaler and Purcell, 2019; Sadeqi et al., 2025).

#### Gene annotations

The gene annotation for all significant SNPs detected under both conditions was identified using the Ensembl Plants database (Bolser et al., 2016). The International Wheat Genome Sequencing Consortium (IWGSC) Reference Sequence v1.0 was used to find out the physical position of SNPs resulting from genotyping-by-sequencing (GBS), while the flanking sequences of SNP markers resulting from 25K were obtained from the GrainGenes database (https://wheat.pw.usda.gov/GG3/). Then, the physical positions (GBS set) and flanking sequences (25K set) were blasted into the Ensembl database to identify the candidate genes and their functional annotations. The candidate genes were selected if significant SNPs were located within them.

#### Gene network analysis

The genetic network among all genes detected under drought stress by gene annotation was further investigated. Functional gene enrichment of these genes was investigated using the ShinyGo 0.82 database (Ge et al., 2020), focusing on the molecular function (MF), cellular component (CC), biological process (BP), and Kyoto Encyclopedia of Genes and Genomes (KEGG) pathways. A false discovery rate with a *p*-value of <0.11 was applied as a threshold to identify significantly enriched pathways. The same flexibility threshold was previously used to detect enriched pathways for other traits in wheat.

### Data analyses

Analysis of variance (ANOVA) for all traits measured under control and drought (20% PEG) conditions was conducted with PLABSTAT (Utz, 2011) and the R statistical environment (R Core, 2025) using the linear model:


$$Y_{\left(ijk\right)}\;=\;\mu \;+\;g_{i}\;+\;r_{j}\;+\;t_{k}\;+\;{\left(gt\right)}_{ik}\;+\;\epsilon_{ijk},$$

where Y_(ijk)_ is the phenotypic value of the ith genotype in the jth replication and kth treatment (control or 20% PEG); μ denotes the overall mean; g_i_, r_j_, and t_k_ are the fixed effects of genotype, replication, and treatment, respectively; (gt)_ik_ represents the genotype × treatment interaction; and ϵ_ijk_ is the residual term (genotype × replication × treatment).

The broad-sense heritability was computed using the software PLABSTAT via the HERT command (Utz, 2011). Phenotypic correlation, indicating that it has the highest values for G% (100) on coefficients, was also calculated in PLABSTAT and classified as low (*r* < 0.40), moderate (0.40 ≤ *r* ≤ 0.60), or high (*r* > 0.60).

### Identification of the best genotypes and the worst genotypes

The MGIDI was applied to select for the best genotypes having the highest performance under drought, and the selection was conducted according to Olivoto and Nardino (2021). The best 10 and the worst 10 genotypes were identified using the classification matrix of Olivoto and Nardino (2021).

All figures and illustrations were generated using the R package software 4.5.1 (R Core, 2025) and the SRplot data visualization (Tang et al., 2023).

## Results

### Analysis of variance and correlations

**Figure 1:**
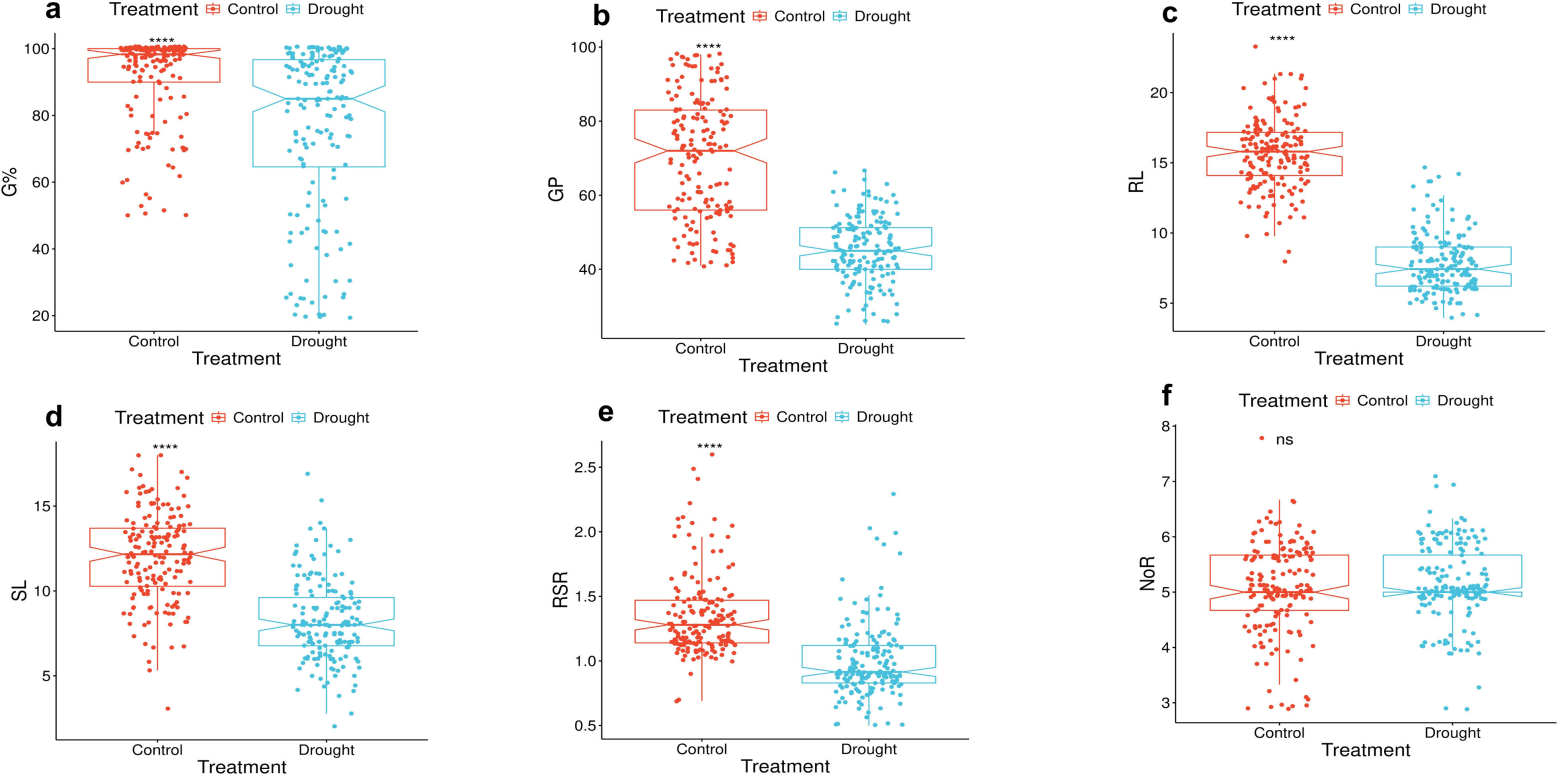
Box plot of traits in 168 wheat genotypes under control (0% PEG) and drought (20% PEG).; **(a)** G%, germination percentage; **(b)** GP, germination pace; **(c)** RL, root length; **(d)** SL, shoot length; **(e)** RSR, root/shoot ratio; **(f)** NoR, number of roots.

The imposed drought stress reduced all traits compared to the control treatment (**Figure 1**). The reduction was very pronounced in the GP, RL, SL, and RSR (**Figures 1B–E**). Meanwhile, the germination percentage (G%) and NoR showed less reduction (**Figures 1A, F**). The ANOVA conducted under control (0% PEG) and drought stress (20% PEG) conditions revealed highly significant differences (*p* ≤ 0.01) among treatments, genotypes, and their interactions (T × G) for nearly all studied traits, including G%, GP, RL, SL, RSR, and NoR, except NoR under the treatment effect, which was non-significant (**Table 1**). These findings indicate strong and significant genotype-specific responses to drought. Heritability estimates for the traits ranged from 85% to 99%. All traits showed high heritabilities, G% (99%), GP (98%), RL (95%), SL (97%), RSR (96%), and NoR (85%) (**Table 1**). Such high heritability estimates indicate that the multi-trait selection strategy can be conducted in this wheat collection.

**Table 1:** Analysis of variance (ANOVA) and heritability in wheat genotypes under control (0% PEG) and drought (20% PEG) conditions.

Source of variance	G%	GP	RL	SL	RSR	NoR
Treatments	182.85**	763.07**	1,005.16**	226.89**	128.10**	1.85
Replications	1.14	0.17	1.62	0.26	0.61	0.90
Genotypes	445.23**	104.62**	29.85**	62.93**	41.29**	9.03**
Treatment × genotype	68.74**	25.28**	25.32**	44.30**	32.68**	5.33**
Heritability	0.99	0.98	0.95	0.97	0.96	0.85

*, **, and *** indicate significance levels at *p* ≤ 0.05, 0.01, and 0.001, respectively.

**Figure 2:**
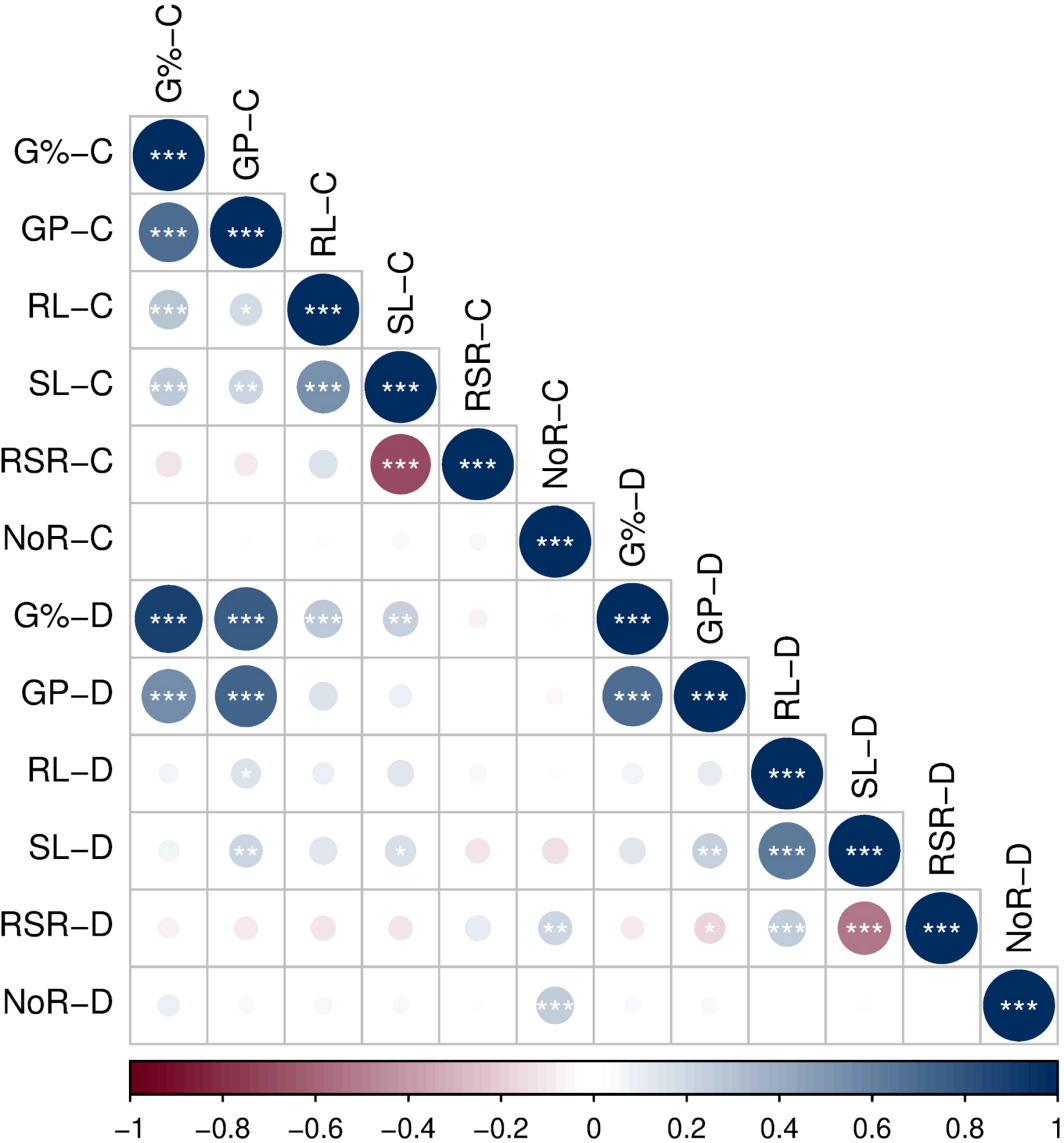
Correlation of traits in 168 genotypes under control (0% PEG) and drought (20% PEG).

Correlations among each pair of the traits varied from low to high under both treatments: control and drought. Under control, G% showed significant correlations (*p* > 0.001) with GP, RL, and SL, with *r* = 0.68***, 0.29***, and 0.27***, respectively. Similarly, RL and SL exhibited moderate and significant correlation with *r* = 0.53***. However, SL and RSR showed a strong negative correlation with *r* = −0.7*** (**Figure 2**). Under drought, similarly, G% and GP exhibited the highest positive correlation with *r* = 0.69***, followed by the correlation between SL and SL with *r* = 0.62***. The negative correlation between SL and RSR lowered to *r* = −0.54*** (**Figure 2**). These results indicate a coordinate control of the germination-related traits G% and GP under the control and drought treatments; the same pattern was observed for the seedling-related traits SL and RL.

### Principal component analysis

**Figure 3:**
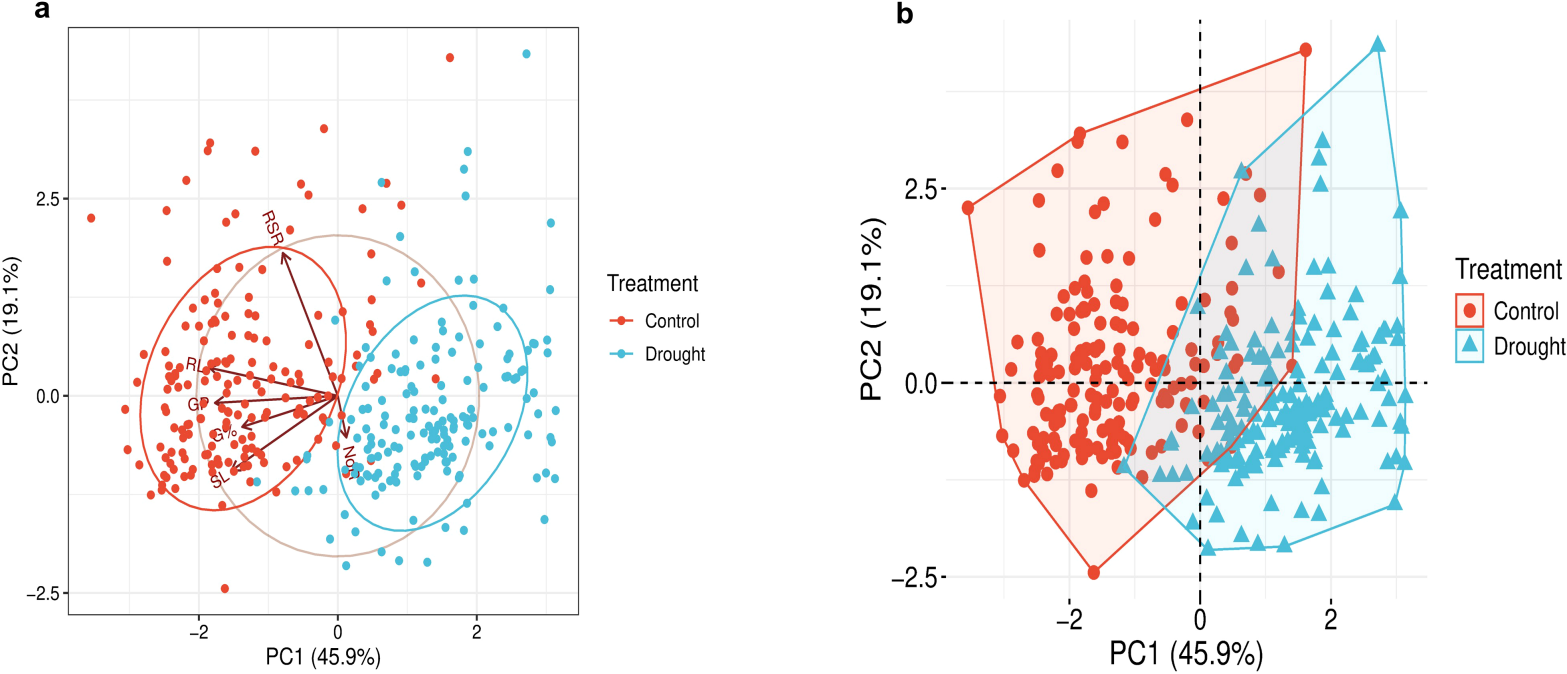
Principal component analysis (PCA) in 168 wheat genotypes under control (0% PEG) and drought (20% PEG) treatments: **(A)** biplot of the traits and **(B)** PCA showing the clustering of genotypes under treatments.

Principal component analysis exhibited a clear discrimination between the control and drought-treated genotypes. Together, PC1 and PC2 accounted for 65% of the total variation. PC1 captured the largest portion of variation, 45.9%, and PC2 accounted for residual variation of 19.1% (**Figures 3A, B**). The control group clustered tightly together in the negative domain of PC1, showing low variance, indicating that the genotypes respond similarly under control conditions. Meanwhile, under drought, the genotypes exhibited a wider distribution range and mainly clustered in the positive domain of PC1, indicating wider variation and extended plasticity under drought stress (**Figures 3A, B**). A low interference between the distribution of the genotypes under both treatments was observed, mirroring the effectiveness of the drought stress on the traits of genotypes under control. The vectors of the traits are oriented to the negative domain of PC2; this pattern reflects the reduction trend of most traits under drought stress, except RSR and NoR. This pattern aligns with the pattern presented in **Figure 1** and supports the correlation patterns presented in **Figure 2**.

### Identification of the best genotypes and the worst genotypes

**Figure 4:**
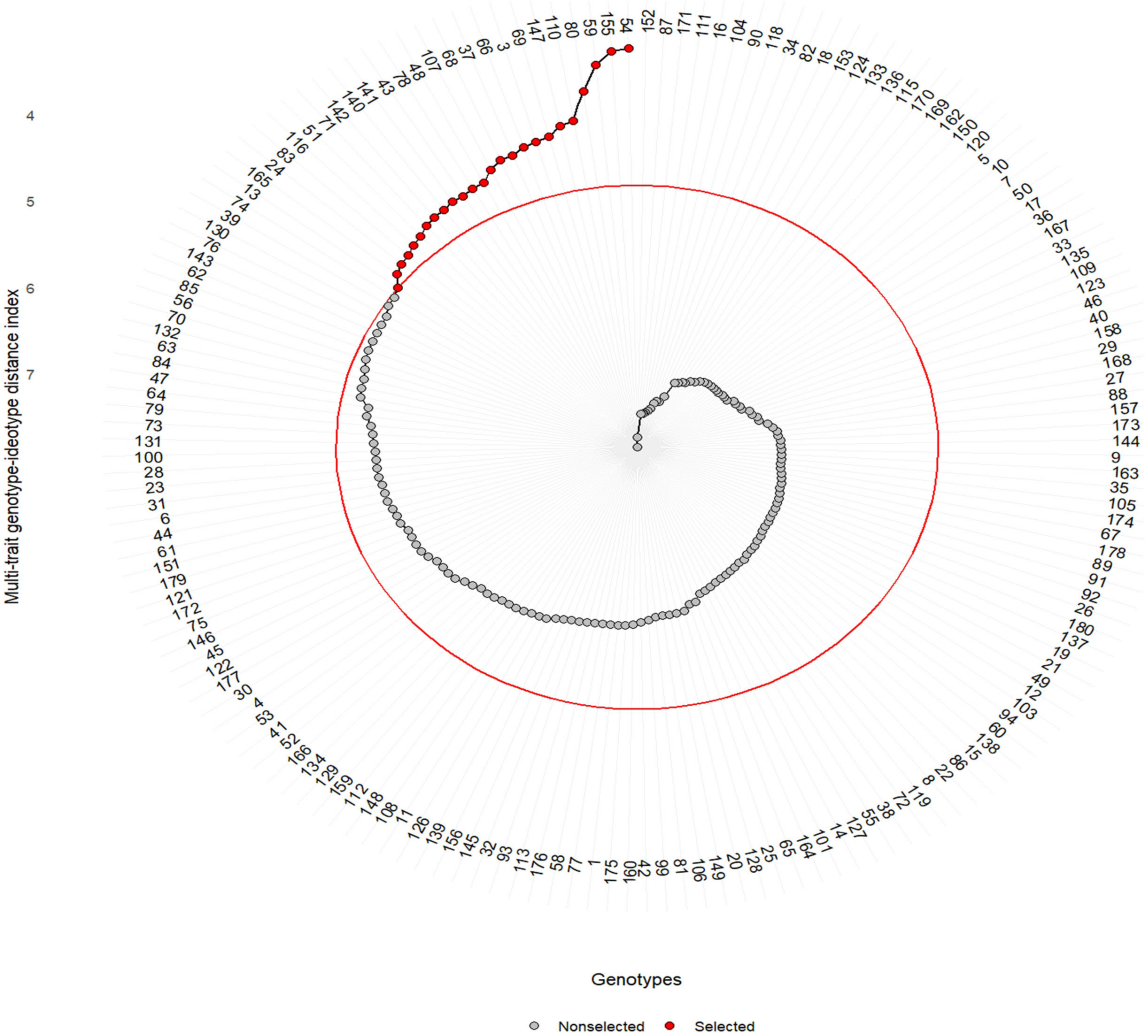
Ascending ranking for the MGIDI index of the genotypes based on their performance under drought. The selected genotypes are marked red, while the non-selected genotypes are marked black. The red circle represents the cut point of selection pressure.

**Figure 5:**
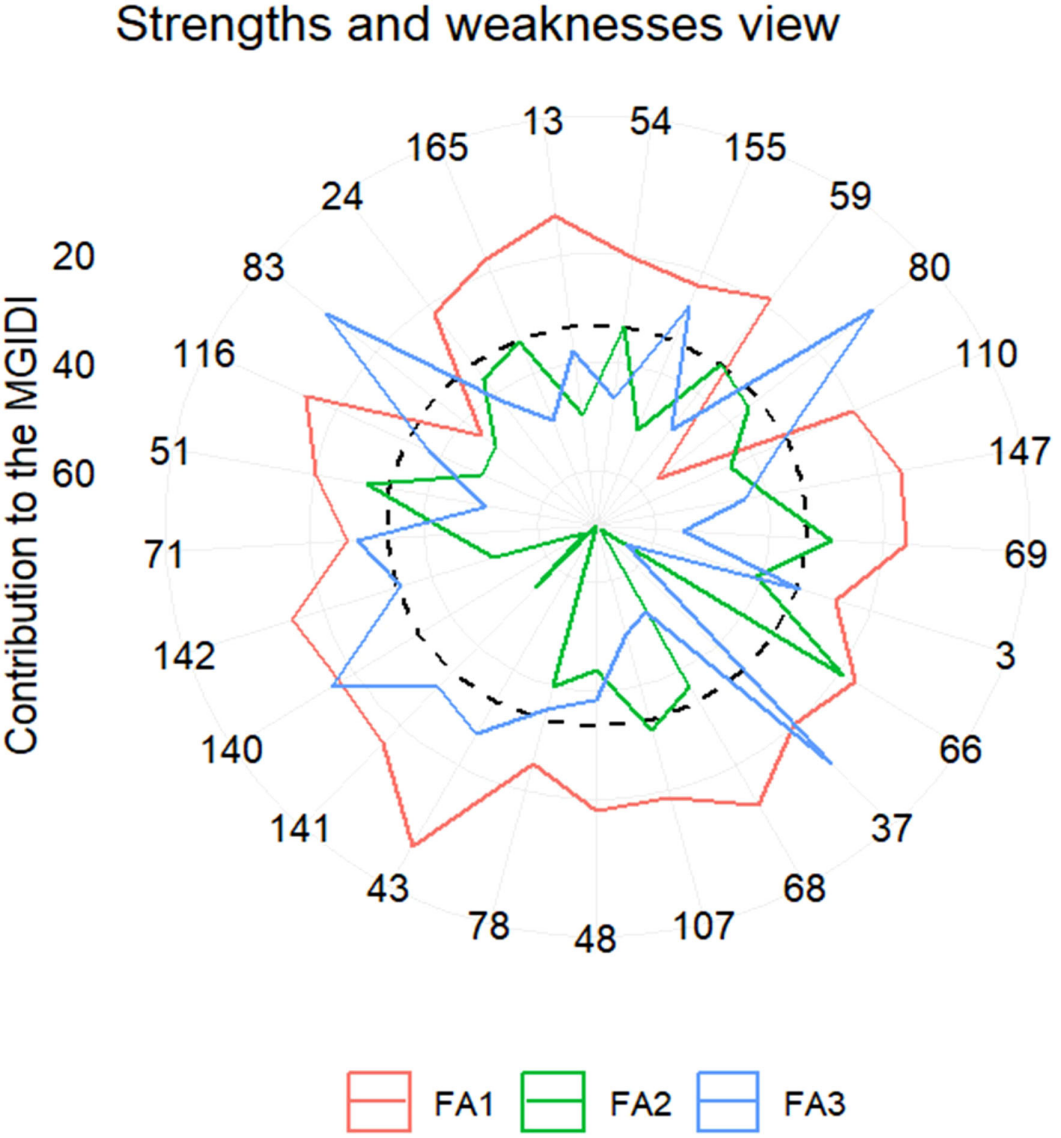
Plot of the strengths and weaknesses of the selected genotypes is shown as the proportion of each factor on the computed multi-trait genotype–ideotype distance index (MGIDI). FA1 includes G% and GP, FA2 includes SL and RL, and FA1 includes RSR and NoR. The dotted line represents the theoretical value if all the factors had contributed equally.

**Figure 6:**
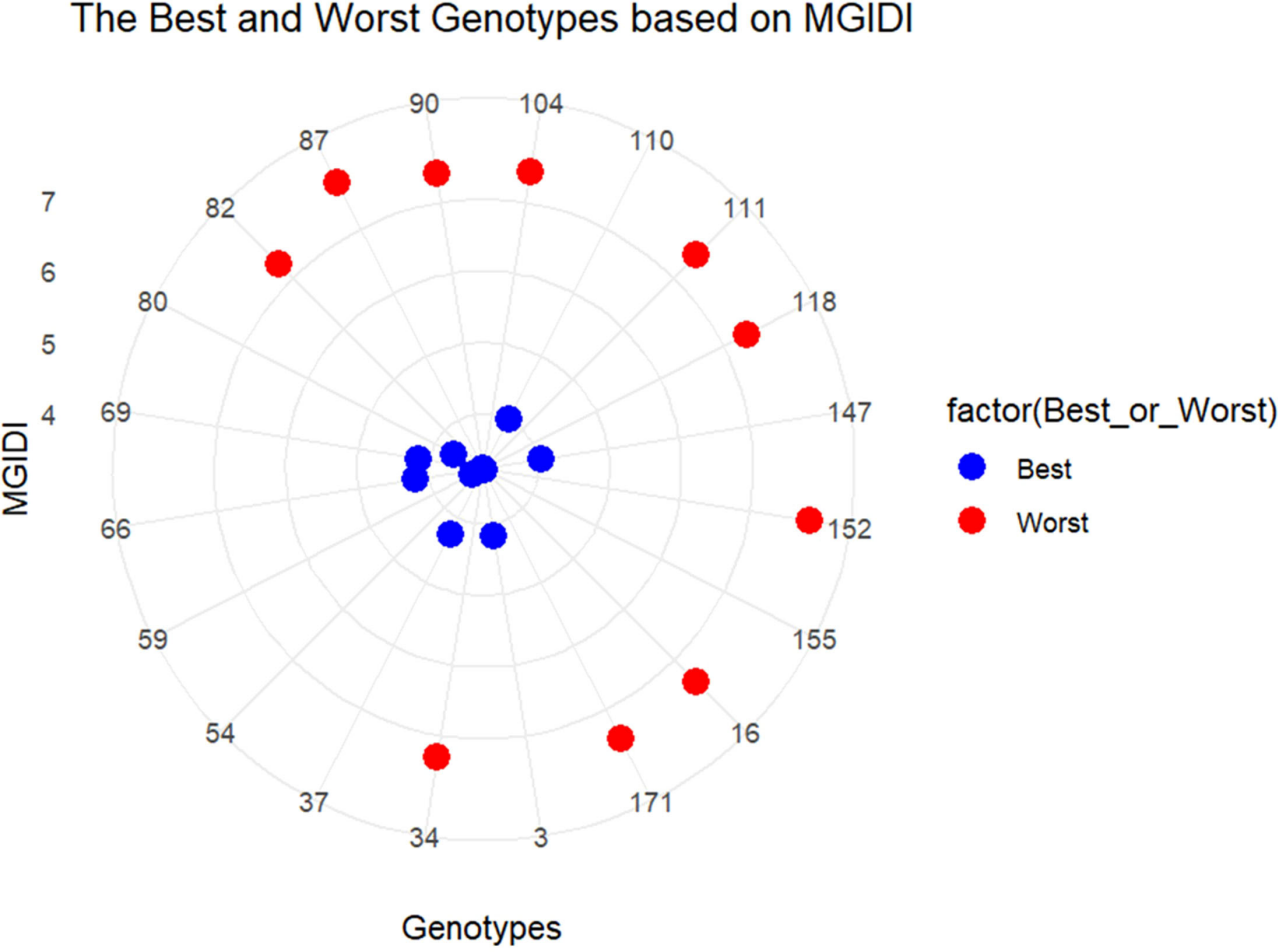
The distance between the best 10 genotypes and the worst genotypes relative to the idiogenotype. The best genotypes are labeled blue and the worst are labeled red.

Given that the heritability estimates were high, ranging from 0.85 to 0.99 (**Table 1**), a multi-trait approach was applied to detect the best genotypes using the MGIDI, in which, based on the ultimate performance of each genotype, the distance between each genotype and a computed ideotype was calculated. A set of 25 genotypes was identified as the best genotypes based on their performance under 20% PEG drought stress (**Figure 4**). **Figure 5** shows the clustering of the six traits in three factors, G% and GP clustered in the first factor (FA1), SL and RL grouped in the second factor (FA2), and RSR and NoR separated in the third factor (FA3). The strengths and weaknesses of the best 25 genotypes, contributed by each factor to their MGIDI index, are shown in **Figure 5**. FA1 had the lowest contribution for genotype 43, indicating that it has the highest values for G% (100%) and GP (61). On the other hand, FA1 had the highest contribution of the MGIDI of genotype 80, implying that it has the lowest G% (63.3%) and GP (34) (data not shown). FA2 showed the lowest contribution in the MGIDI of genotype 66 (RL = 14 cm, SL = 15.3 cm), while it showed the highest contribution for the MGIDI of genotype 37 with RL = 9.3 cm and SL = 4.6 cm (data not shown). FA3 revealed the lowest contribution in the MGIDI of genotype 37 with RSR = 2.03 and NoR = 7. Contrarily, FA3 showed the highest contribution in the MGIDI of genotype 66 with RSR = 0.9 and NoR = 4 (data not shown). Among the best 25 genotypes, the best 10 genotypes have the lowest distance values from the computed ideotype, ranging from 3.2 for the genotype coded 54 from Algeria to 4.2 for the genotype coded 37 from Sudan (**Supplementary Table 1**; **Figure 5**), while the most drought-sensitive 10 genotypes had distance values, ranging from 7.2 for the genotype coded 82 from Ethiopia to 7.8 for the genotype coded 152 from Saudi Arabia (**Supplementary Table 1**; **Figure 6**).

### Genome-wide association study

GWAS was performed between all traits scored under both conditions and all SNP markers generated from the 25K SNP array. The details of GWAS for the significant markers detected under both conditions are presented in **Supplementary Table 2**. Based on the Q-Q plot results (**Supplementary Figures 1**, **2**), the FarmCPU model was fit with most of the traits. The significant markers detected under drought (130 SNPs) were slightly higher than those detected under normal conditions (128 SNPs). Chromosome 5B had the highest number of significant SNPs under control conditions, while the highest number of significant SNPs was found in chromosome 5A under drought conditions. According to the wheat genomes, a total of 65 and 63 significant SNPs were found in genomes B and A under normal and drought conditions, respectively (**Supplementary Figure 3**). A set of 41 significant markers was found to be associated with RSR under control, while 48 significant SNP markers were associated with NOR under drought. The gene annotation was investigated for each marker (**Supplementary Table 3**). Under controlled conditions, 106 different genes were identified, while 98 different genes were detected under drought stress. Notably, it was found that different markers were located within the same gene model. For example, *Kukri_c12562_453*, *RFL_Contig570_515*, and *wsnp_Ex_c10644_17356566* were located within the same gene model (*TraesCS5B02G292200*) under control conditions. Out of the 98 genes detected under drought conditions, 54 had an association with drought tolerance (**Supplementary Table 4**). Moreover, five genes had an association with drought tolerance and germination rate.

**Figure 7:**
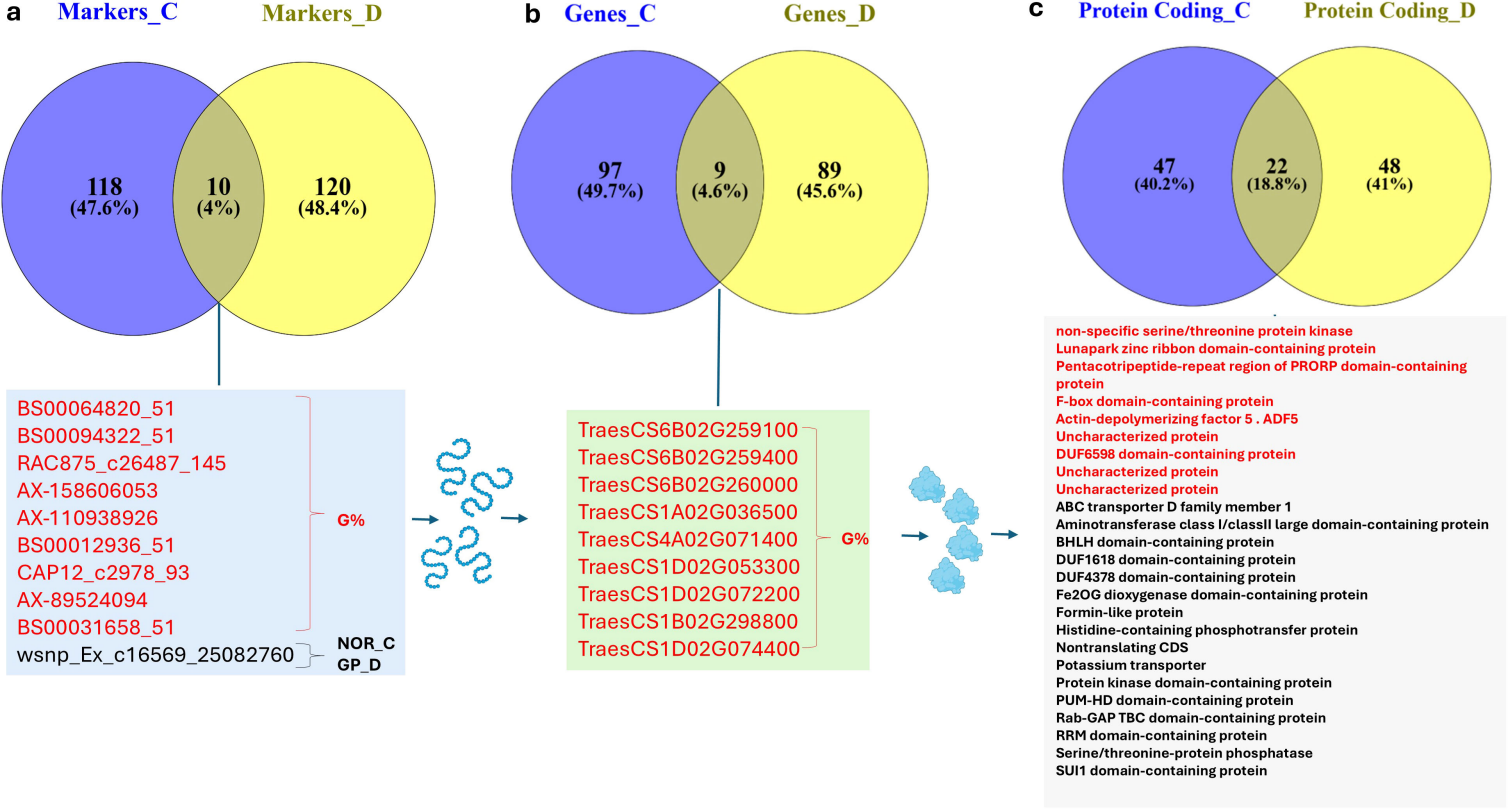
Venn diagram shows the common and specific factors between control (0% PEG) and drought (20% PEG): **(A)** common SNP markers, **(B)** common genes, and **(C)** common protein-coding genes. C stands for control and D stands for drought.

Interestingly, 10 significant markers were shared between the control and drought stress (**Figure 7A**). Out of the 10 significant markers, nine were found to be associated with germination percentage (G%) under both conditions (**Figure 7A**). The other left marker was associated with GP_D and NOR_C. The 10 markers were located on 1A (one), 1B (one), 4A (one), 1D (three), and 6B (three). The common genes detected under both conditions were investigated (**Figure 7B**). Nine gene models were shared between the control and drought stress. Nine genes were associated with G%. Moreover, 22 coding proteins were shared between the control and the drought stress conditions (**Figure 7C**).

### Gene network analyses

The gene network analyses of the nine common genes and all genes were investigated. Gene-specific network analysis indicated that four of the nine genes have a direct role in drought tolerance during seed germination, as they interact with multiple downstream genes involved in drought-responsive processes. These four genes interact with more genes to confer their effects. These genes are *TRAESCS1D02G072200* (12 interacting genes), *TRAESCS1D02G074400* (3 interacting genes), *TraesCS1B02G298800* (3 regulator genes), and *TRAESCS6B02G260000* (12 interacting genes). The remaining three genes and their interactive genes were *TraesCS1A02G036500* (6 interacting genes), *TRAESCS1D02G074400* (10 interacting genes), and *TraesCS6B02G259400* (8 interacting genes), and they showed an indirect association to drought tolerance by regulating traits such as lateral roots and seed imbibition. Meanwhile, the last two genes *TRAESCS6B02G259100* (5 interacting genes) and *TRAESCS4A02G071400* (13 interacting genes) were not involved in drought tolerance (**Supplementary Figure 3**).

**Figure 8:**
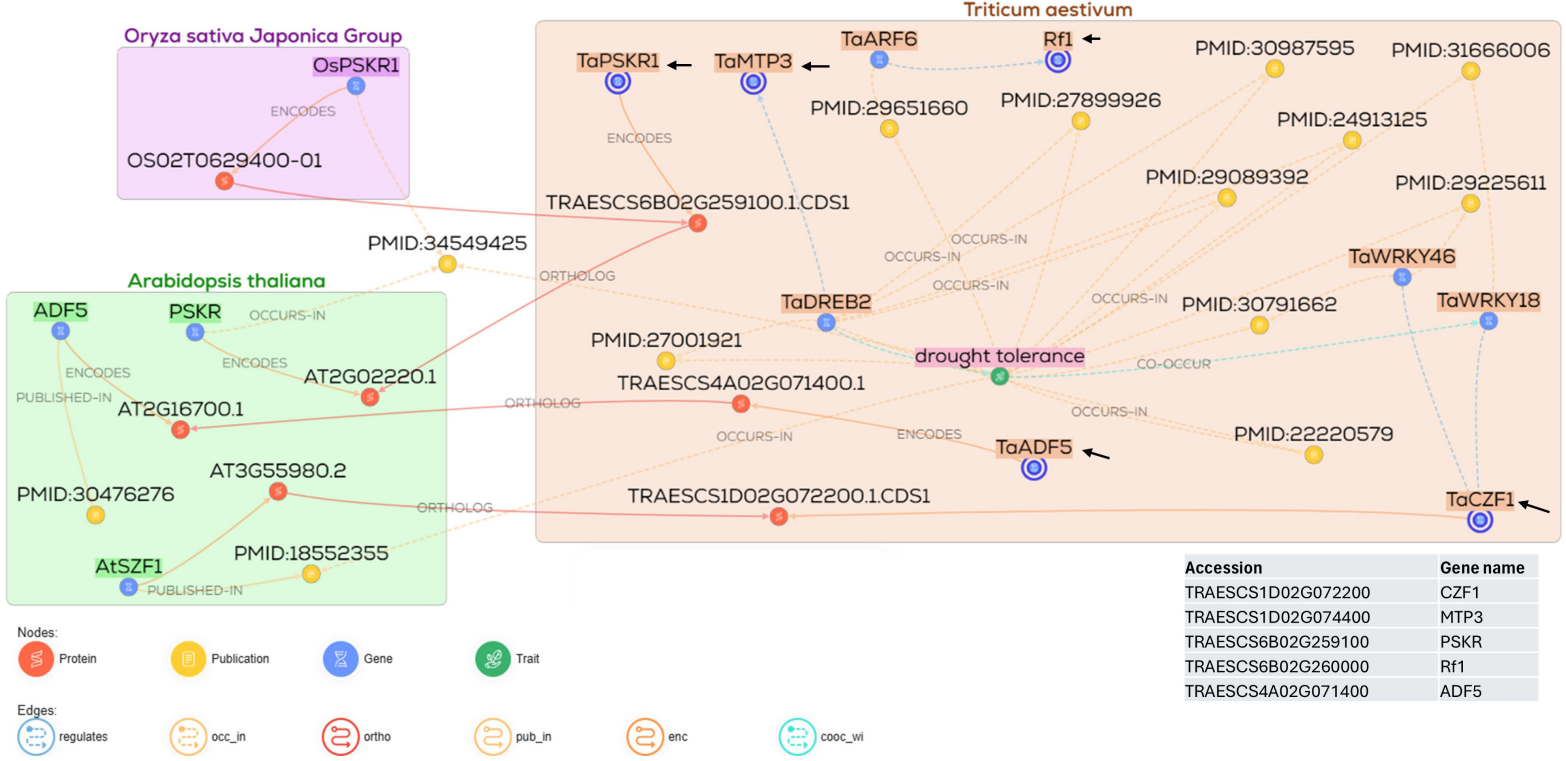
Gene network of nine gene models, showing the gene–gene interactions under drought stress in wheat and their respective orthologs in rice and *Arabidopsis*.

The gene network analysis for all the nine genes associated with G% is illustrated in **Figure 8**. Among the three genes, *TRAESCS1D02G072200* (*CZF1*) gene encodes a CCCH-type zinc finger protein that plays a role in drought tolerance in plants.. The *CZF1* gene encodes a CCCH-type zinc finger protein that plays a role in drought tolerance in plants. *TRAESCS1D02G074400* codes for metal tolerance protein (*MTP3*), which is necessary for ion homeostasis, especially the divalent ions such as Zn and Mn. *TRAESCS6B02G260000* (*RF1*) encodes for the pentacotripeptide-repeat region of PRORP domain-containing protein. *TRAESCS1D02G072200* (*CZF1*) regulates *WRKY18* genes, which are associated with drought tolerance in wheat. *TRAESCS1D02G072200* (*CZF1*) encodes a protein orthologous to *OsC3H35* (*Os05g0195200*) in rice, which encodes the delay of the onset of senescence-like protein reported to confer drought tolerance, and to AT3G55980 encoded by *AtSZF1* in *Arabidopsis*, which has been associated with salinity tolerance. *TRAESCS6B02G259100* that codes for phytosulfokine receptor 1 (*TaPSKR1*) and *TRAESCS4A02G071400* that codes for actin-depolymerizing factors (*ADF5*) did not show any connection with drought tolerance.

**Figure 9:**
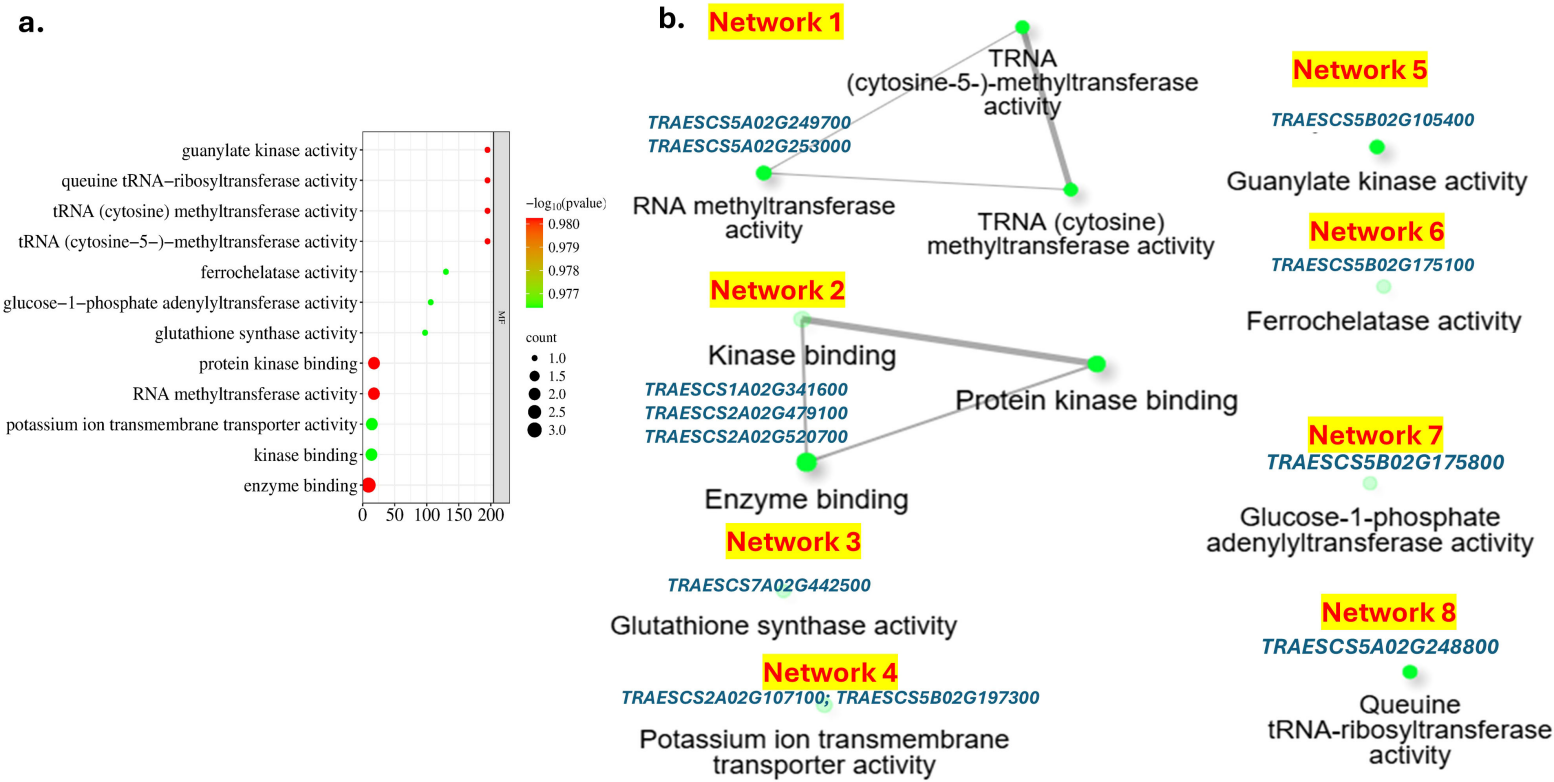
Enrichment analysis of the identified gene models controlling drought tolerance: **(A)** molecular pathways and **(B)** network of the MF pathways and their gene models controlling them.

For the 98 genes associated with drought tolerance, no significant BP, CC, or KEGG pathways were found. Based on MF, 44 pathways were identified (**Supplementary Table 4**). The number of significant pathways was reduced to 12 pathways controlled by 12 gene models based on FDR <0.11 (**Figure 9A**). These genes were found to work together in eight different networks (**Figure 9B**). Networks 1 and 2 contain three different pathways each. Network 1 is controlled by two different genes, *TRAESCS5A02G253000* and TRAESCS5A02G249700, and is associated with RNA and TRNA methyltransferase. On the other hand, network 2 was controlled by three gene models (*TRAESCS1A02G341600*, *TRAESCS2A02G479100*, and *TRAESCS2A02G520700*) and was associated with kinase and protein kinase binding. The remaining six networks contained only one pathway for each one and were controlled by one gene model, except network 7, which was controlled by two genes associated with potassium transmembrane transporter activity.

## Discussion

Drought decreased all traits compared to the control. The reduction was pronounced in all traits except RSR and NoR. These results imply the inhibitory effect of 20% PEG on seed germination and seedling vigor. The same concentration was previously used in investigating drought tolerance in the bread-wheat diversity panel at germination stages (Mohamed et al., 2023; Ahmed et al., 2025). It was reported that 20% PEG-6000 corresponds to an osmotic potential commonly used to impose moderate-to-severe drought stress during germination assays in wheat (Ahmed et al., 2025). The highly significant effects of genotype, treatment, and their interactions mirror the role of genetic factors underlying the observed genotypic variation across all traits. These results indicate that drought resilience is a genotype-dependent trait that is influenced by the environment. In an 80-bread-wheat collection, Ahmed et al. (2025) found that phenotypic variation for all traits was attributable to significant effects of genotype, treatment, and their interaction. The high heritability estimates observed in the current study (0.85 to 0.99) suggest that multi-trait selection may be applicable to selecting the best drought-resilient genotypes. In the current study, highly significant correlations were observed between the traits under control and drought (**Figure 2**). These high correlations between SL and RL imply that plants favor a balanced allocation of resources in the root and shoot organs. Additionally, the germination-related traits (G% and GP) exhibited the same trend, indicating a synchronous regulation of germination performance traits.

These high heritabilities concur with the 0.55–0.99 range observed for the same traits under 20% PEG in a 198-genotype wheat panel (Gudi et al., 2024). Similarly, high heritabilities were observed in the same traits under 25%–30% PEG (89.4%–98.7%) in a 41-genotype spring-wheat collection (Mohamed et al., 2023). These high heritabilities even under drought conditions suggest strong additive genetic control that facilitates the selection of drought-resilient genotypes. These traits represent reliable targets for selection, especially when integrated with marker-assisted or genomic tools focused on drought-responsive loci. This notion is also supported by the highly significant correlations observed between these traits, particularly under 20% PEG treatment (**Figure 2**).

In accordance with our results, for both genotypes and traits, PCA successfully described the shifts of seed germination and seedling vigor under drought and distinguished the drought-stressed genotypes from the non-stressed ones in several cereals. In bread wheat, PC1 accounted for 35.4% of the total variation of G%, RL, SL, root fresh weight, and NoR, clearly differentiating the tolerant from the sensitive lines (Mohi-Ud-din et al., 2021). In tetraploid wheat, PCA of G%, RL, SL, and NoR contributed to classifying the drought-stressed genotypes into distinct tolerance groups (Benlioğlu et al., 2024; Sallam et al., 2026b). Recently, Ahmed et al. (2025) found that in 80 genotypes of wheat, PC1 explained 94% of the total variation of G%, SL, and RL and obviously ranked the drought-resilient genotypes. Similar results were reported in other cereals, including rice (Nyasulu et al., 2024), barley (Song et al., 2024), and sorghum (Wang et al., 2025). As a validation of our results, we found that among the 25 superior genotypes identified in the current study, the genotypes Beni-Suef-5 and Sakha93 were classified as drought-tolerant by Ahmed et al. (2022). Similarly, all the sensitive genotypes identified in the current study were ranked as sensitive in their study; however, in their study, other traits and a selection matrix were used in both studies.

The 10 most drought-tolerant genotypes showed the lowest MGIDI index values and showed the closest distances to the computed ideotype (**Figures 4**, **6**). The MGIDI index was successfully used to identify the superior top-performing genotypes in various crop species. In wild wheat, superior drought-tolerant genotypes were selected among 146 genotypes at early stages (Pour-Aboughadareh and Poczai, 2021). Also, in wheat, MGIDI successfully ranked and classified a set of 14 tolerant genotypes into 13 tolerant and 1 sensitive genotype (Mohi-Ud-Din et al., 2025). Similarly, MGIDI was used to identify the top-performing genotypes for waterlogging in sesame at the pre-flowering stage (Ahsan et al., 2024). The MGIDI index was employed to select superior black oat genotypes based on agronomic traits (Klein et al., 2023). Recently, Salami et al. (2025) identified and ranked the most drought tolerant genotypes in rapeseed.

### Genome-wide association study and QTL network analysis under drought stress

The GWAS was performed across all traits under both conditions and SNP markers, revealing important SNPs, with the majority of them falling within candidate genes. This was expected given the high genetic variation among genotypes for all traits. The same population was previously used to identify QTLs associated with drought tolerance at the seedling stage (Sallam et al., 2024b), drought tolerance at the adult growth stage, salt tolerance at both germination and vegetative stages, disease resistance (Esmail et al., 2023; Mourad et al., 2023b, 2025), and heavy metal tolerance (Mourad et al., 2023a; Sharshar et al., 2025). The significant markers were detected at *p ≤*0.001, which is less conservative than the false discovery rate and Bonferroni correction. Using very stringent significance thresholds, such as Bonferroni- or FDR-adjusted *p*-values, can result in overlooking informative markers/genes with small effects. In GWAS, a *p*-value threshold of 0.001 is often adopted as a more permissive yet still acceptable criterion for detecting potential candidate associations (Sallam et al., 2026a). However, functional validation in a different genetic background remains essential to validate the true association.

A set of nine GWAS models, including PCA, kinship, and PCA + kinship, was used to correct the effect of population structure, which could cause a spurious association. The population structure of this material was previously investigated in detail using SNP markers generated by a 25K SNP array (Sallam et al., 2024b) and GBS (Mourad et al., 2020).

The highest number of significant SNPs under drought stress was found in genome A, suggesting that genome A may include multiple genes that influence drought tolerance due to the presence of multiple genes. Therefore, investigating the specific genes and mechanisms controlling these QTL will provide valuable information for developing drought-tolerant wheat cultivars. The predominance of significant SNPs within the B and A genomes, consistent with their large size and gene density in wheat, further supports the importance of these genomes in regulating both baseline and stress-responsive traits; moreover, Chr. 5A had the highest number of QTL detected under drought stress. It was also found that 5A included the highest number of QTL under drought stress (Kumar et al., 2020; Puttamadanayaka et al., 2020; Cabas-Lühmann et al., 2024).

The gene annotation analysis confirmed the GWAS results performed in this study. In total, 98 genes were identified to be associated with all traits. Interestingly, a set of 54 genes was previously reported to be linked to drought tolerance in wheat, indicating that the GWAS performed in this study precisely identified true associations with drought tolerance. The remaining 44 genes could be novel genes associated with drought tolerance. GWAS is considered a powerful approach for identifying novel genes associated with target traits, especially those that are polygenic (e.g., drought tolerance). Novel genes identified in the current study may be tested for gene expression to validate their association with drought tolerance. Among the 54 genes, five were found to be associated with germination rate and drought tolerance. For example, *TRAESCS5B02G175800* encodes glucose-1-phosphate adenylyltransferase, which is known as ADP-glucose pyrophosphorylase (AGPase). AGPase, as the key enzyme in starch synthesis, is crucial for maintaining this buffering capacity. It helps plants cope with drought stress in several ways: maintaining energy balance, protecting cellular processes (Dai et al., 2023), and facilitating osmotic adjustment (Huan et al., 2022). The same enzyme plays an important role in the biosynthesis process of starch, which is necessary for providing energy and building blocks for the developing seedling (Pontis, 2017). Bearing in mind that the gene *TRAESCS5B02G175800* had a marker that was found to be associated with the shoot/root ratio under drought stress,. Therefore, this finding further supports the GWAS analysis in identifying the true candidate genes for target traits.

Interestingly, among all significant markers, only 10 markers were significant under both conditions, most of which (nine markers) were linked to germination percentage (G%). This limited overlap implies that the majority of genetic determinants for these traits are condition-specific, although a small set of stable loci may be expressed across environments in a constitutive manner. Loci detected in both control and drought conditions resemble the “constitutive” or “stable” QTL/SNPs. Similar results were reported in rapeseed, barley, and rice, which affect germination and early growth across environments and often show pleiotropic effects on core vigor or growth traits (Moursi et al., 2020; Gad et al., 2021; Hasseb et al., 2022; Slawin et al., 2024; Yang et al., 2024). Such environmentally stable loci are valuable for breeding because they contribute to robust establishment under variable field conditions, while stress-specific loci can be targeted to enhance drought tolerance at germination (Vargas et al., 2006; Collins et al., 2008; Hao et al., 2010; Moursi et al., 2014; Hasseb et al., 2022). The small set of shared SNPs superimposed on many condition-specific associations is biologically expected and supports a dual genetic architecture of early drought tolerance: a broad, inducible stress-responsive component layered on a smaller, constitutive vigor framework.

The nine markers were found to be located within nine different genes. Three of these genes encode uncharacterized proteins. *TRAESCS1D02G072200* (*TaCZF1*) encodes a CCCH-type zinc finger protein that plays a role in drought tolerance in plants. The role of this gene in drought tolerance was demonstrated; its overexpression enhanced drought tolerance and improved root growth in *Arabidopsis* (Chen et al., 2024). Supportive of our findings, the expression of *OsTZF1* (a CCCH-type zinc finger protein) in rice was induced by drought, salinity, and H_2_O_2_ and conferred drought tolerance by controlling the stress-related genes (Jan et al., 2013). The induction of the CCCH-type zinc finger protein-coding gene IbC3H18 enhanced the tolerance to abiotic stresses, including NaCl and PEG-6000, in sweet potato (Zhang et al., 2019). In agreement with our gene network analysis, the IbC3H18 acted as a nuclear transcriptional regulator that regulated the expression of several abiotic stress-responsive genes, including reactive oxygen species (ROS) scavenging, ABA signaling, and ion transport pathways. The interaction of IbC3H18 with the IbPR5 (a disease resistance gene) resulted in the overexpression of IbPR5, which enhanced the salt and drought tolerance in transgenic tobacco plants (Zhang et al., 2019). Additionally, the CZF1 ortholog in *Arabidopsis* (a CCCH-type zinc finger protein) has been involved in salt stress and immune responses. These results indicate that the CCCH-type zinc finger proteins are abiotic/biotic stress-responsive factors.

*TRAESCS6B02G260000* (*RF1*) encodes for the pentacotripeptide-repeat region of the PRORP domain-containing protein. The pentacotripeptide-repeat proteins modulated the mitochondrial RNA transcript modification, which is necessary for the proper function of the mitochondria, energy production, and stress resistance (Li et al., 2025a). The *RF* genes were reported with their association with drought stress (Kumar et al., 2018). The pentacotripeptide-repeat coding genes regulated drought tolerance in rice (Peng et al., 2025). In the current study, this gene was found to be associated with G% under control and drought conditions, indicating its constitutive nature and its importance for seed germination.

*TRAESCS1D02G074400* (*TaMTP3*) encodes a metal tolerance protein also known as cation diffusion facilitator (CDF). These proteins were published to play a crucial role in maintaining the homeostasis of microelements in wheat, as well as for biofortification (Vatansever et al., 2017). Similar results were reported in *Triticum urartu*, the donor of the “A” genome of the *Triticum aestivum* (Wang et al., 2021). In the current study, this gene was found to be associated with G%. The orthologs of this gene (MTP8) in *Arabidopsis* maintained Mn homeostasis and Fe remobilization, very essential for efficient seed germination under harsh environmental conditions. Also, this gene can be targeted as a means for biofortification to increase the micronutrient content of the seeds (Eroglu et al., 2017). These three genes were found to be abiotic stress-responsive genes and showed varied expression patterns under drought, heat, salinity, and their combinations (Da Ros et al., 2023).

The nine genes represented different gene networks (**Figure 7**). In the context of our study, the number of stable genes between the two treatments is limited, and the aim of pathway enrichment is clearly exploratory rather than confirmatory. Under these conditions, using FDR < 0.11 is consistent with the recommended practice for discovery-phase analyses and allows us to capture additional candidate pathways while maintaining a quantified upper bound on the expected proportion of false positives (Ji et al., 2011; Chen et al., 2021). These nine stable markers should be validated in different genetic backgrounds for marker-assisted selection. Also, the expression of genes encoding uncharacterized proteins may be useful to confirm their association with drought tolerance in wheat at the germination stage.

Furthermore, the 12 MF pathways and their controlling gene models are good sources to improve drought tolerance in wheat. These genes encode several important proteins: RNA methyltransferase was reported previously as an important process to enhance and regulate drought tolerance in wheat (Shamloo-Dashtpagerdi et al., 2023; Pan et al., 2024) and other plants (Lu et al., 2020). Protein kinase was also reported to be among the factors that regulate the transcriptional response to ABA, a reaction that helps in increasing leaf stomatal closure under drought conditions in wheat leaves (Qiao et al., 2024). Other protein kinase genes were found to enhance drought stress tolerance by facilitating ROS scavenging in *Arabidopsis* (Dong et al., 2025). Additionally, the MF pathways encompassed several ion transporters and regulators. Regulation of potassium transportation across the cell membrane is critical to drought tolerance in many plants such as wheat and barley (Cai et al., 2019; Run et al., 2022; Liu et al., 2025a). Regulation of glutathione homeostasis was reported as an important component that enhances plant tolerance to different abiotic stresses such as drought (Labudda and Azam, 2014; Hasanuzzaman et al., 2017). Therefore, networks 1, 2, 7, and 8 have a direct relationship with enhancing drought tolerance in wheat.

No direct association was reported between the remaining networks and drought tolerance. Nevertheless, the glucose-1-phosphate adenylyltransferase activity pathway identified in network 3 may have an indirect role, as enhanced ADP-glucose pyrophosphorylase (AGPase) activity, especially in the endosperm, has been linked to higher seed yield and biomass, which can provide additional resources to support the plant under stress conditions (V and Tyagi, 2020). Overexpression of ferrochelatase activity, represented in network 4, was reported to improve photosynthetic rates and reduce photo-oxidative damage under drought stress in barley (Nagahatenna et al., 2020). Therefore, ferrochelatase (FC) activity is expected to play an important role in drought tolerance in wheat by influencing the plant’s response to drought and oxidative stress. A significant enrichment in guanylate kinase activity, identified in network 5, under drought stress was reported in maize plants (Li et al., 2025b). However, its role in improving drought stress tolerance in plants is not very clear. No previous studies reported the role of queuine tRNA-ribosyltransferase (QTRT) in drought tolerance in plants. Therefore, its role in improving wheat tolerance to drought is not clear.

Pyramiding these genes together after validation will improve germination rate under drought and normal conditions. The germination percentage in wheat is a critical trait because it directly affects both crop establishment and final yield potential. In wheat breeding, agronomy, and seed quality testing, it is considered one of the core seed performance indicators.

## Conclusion

Drought stress reduced the germination and growth-related traits. GWAS identified the associated genes very successfully. The integration of GWAS and gene network analysis provided deeper insights into drought tolerance in wheat. Several genes interacted and coordinated to confer drought tolerance; these genes include transcription factors and ion transporters. Gene network number 7 was controlled by two genes associated with potassium transmembrane transporter activity, highlighting the potential role of potassium for conferring drought tolerance.

## Data Availability

The SNP datasets generated during the current study are not publicly available due to ongoing research but are available from the corresponding author, Dr. Amira Mourad, upon reasonable request.
