# Supplementary material for: Genome-wide association study and gene network analysis of drought tolerance in wheat during early growth
Source: Front Plant Sci. 2026 Mar 30;17:1775894. doi: 10.3389/fpls.2026.1775894 (PMC13073781; doi:10.3389/fpls.2026.1775894)
Supplement: Supplementary file 2 [file Presentation1.pptx]

## Slide 1
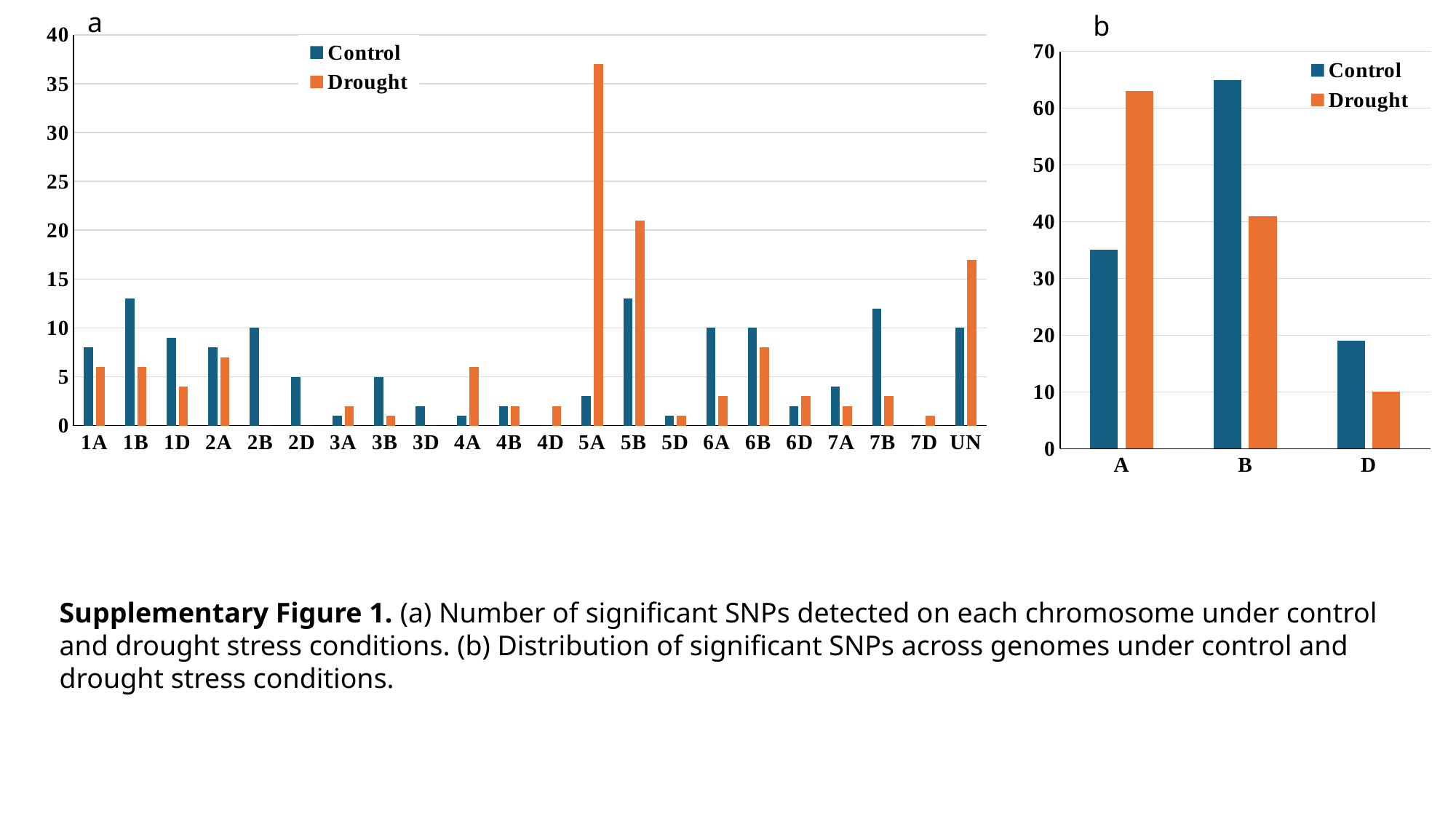

a
b
### Chart
| Category | | |
|---|---|---|
| 1A | 8.0 | 6.0 |
| 1B | 13.0 | 6.0 |
| 1D | 9.0 | 4.0 |
| 2A | 8.0 | 7.0 |
| 2B | 10.0 | 0.0 |
| 2D | 5.0 | 0.0 |
| 3A | 1.0 | 2.0 |
| 3B | 5.0 | 1.0 |
| 3D | 2.0 | 0.0 |
| 4A | 1.0 | 6.0 |
| 4B | 2.0 | 2.0 |
| 4D | 0.0 | 2.0 |
| 5A | 3.0 | 37.0 |
| 5B | 13.0 | 21.0 |
| 5D | 1.0 | 1.0 |
| 6A | 10.0 | 3.0 |
| 6B | 10.0 | 8.0 |
| 6D | 2.0 | 3.0 |
| 7A | 4.0 | 2.0 |
| 7B | 12.0 | 3.0 |
| 7D | 0.0 | 1.0 |
| UN | 10.0 | 17.0 |
### Chart
| Category | | |
|---|---|---|
| A | 35.0 | 63.0 |
| B | 65.0 | 41.0 |
| D | 19.0 | 10.0 |Supplementary Figure 1. (a) Number of significant SNPs detected on each chromosome under control and drought stress conditions. (b) Distribution of significant SNPs across genomes under control and drought stress conditions.

## Slide 2
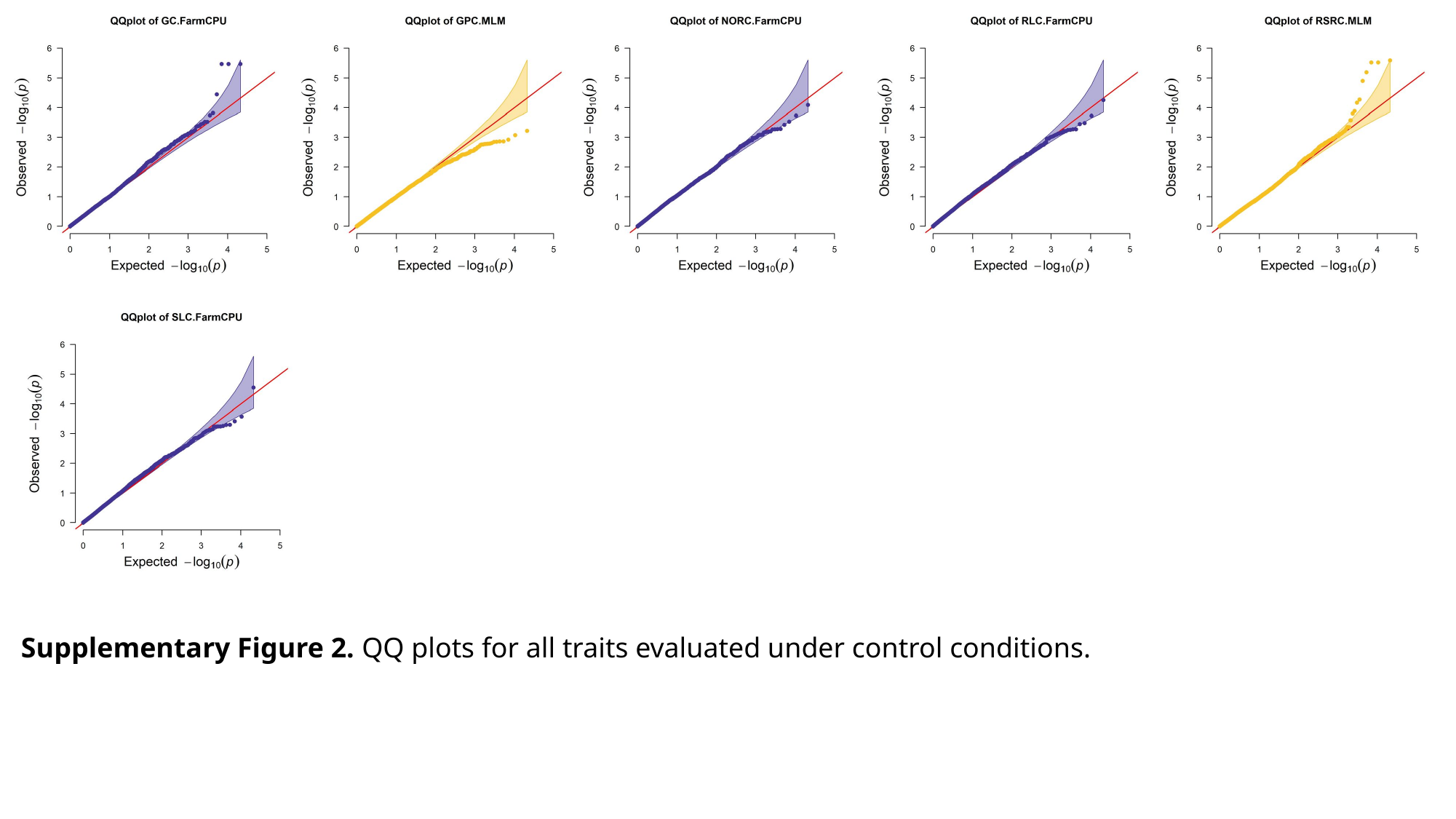

Supplementary Figure 2. QQ plots for all traits evaluated under control conditions.

## Slide 3
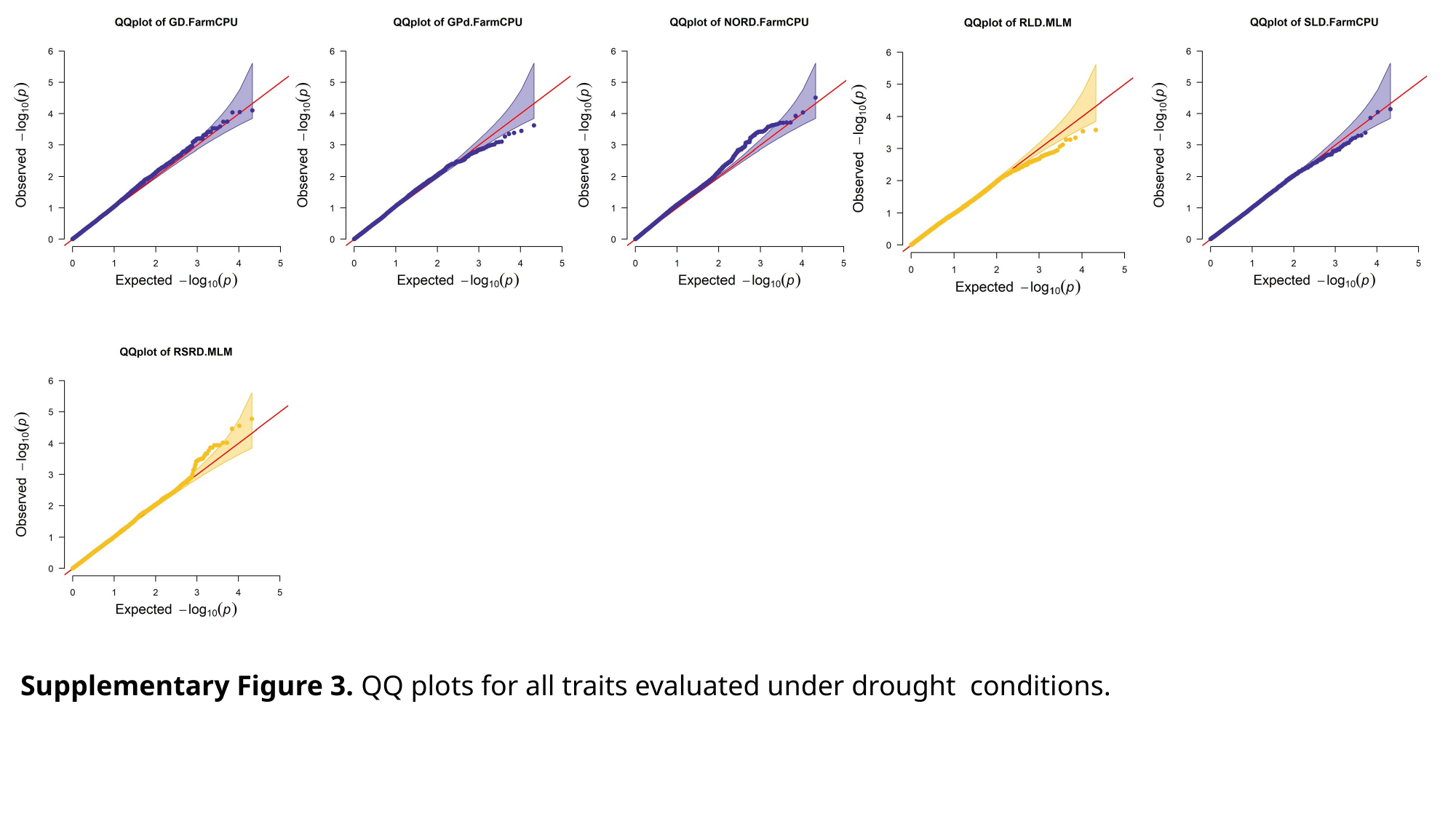

Supplementary Figure 3. QQ plots for all traits evaluated under drought conditions.

## Slide 4
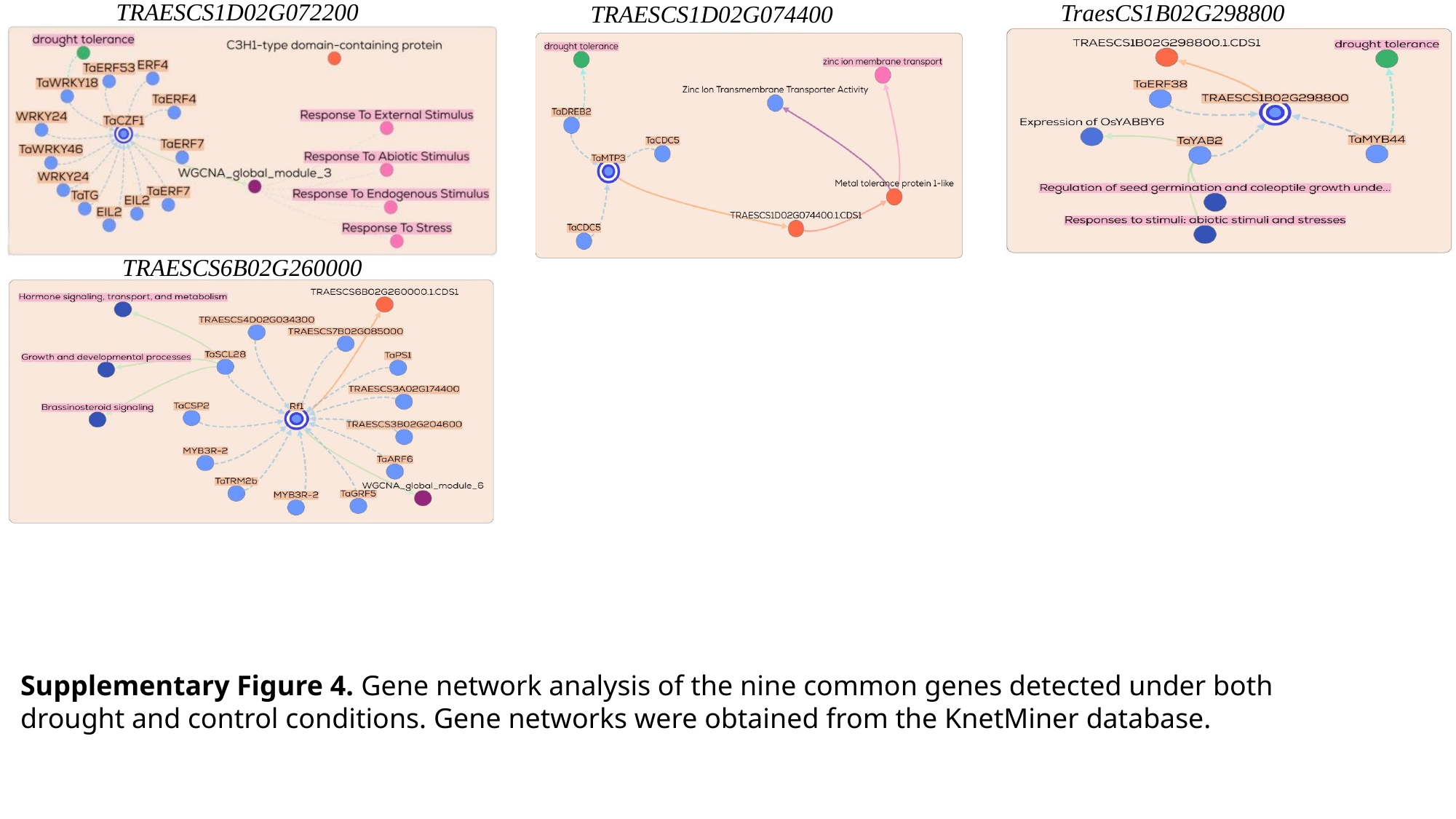

TRAESCS1D02G072200
TraesCS1B02G298800
TRAESCS1D02G074400
TRAESCS6B02G260000
Supplementary Figure 4. Gene network analysis of the nine common genes detected under both drought and control conditions. Gene networks were obtained from the KnetMiner database.

## Slide 5
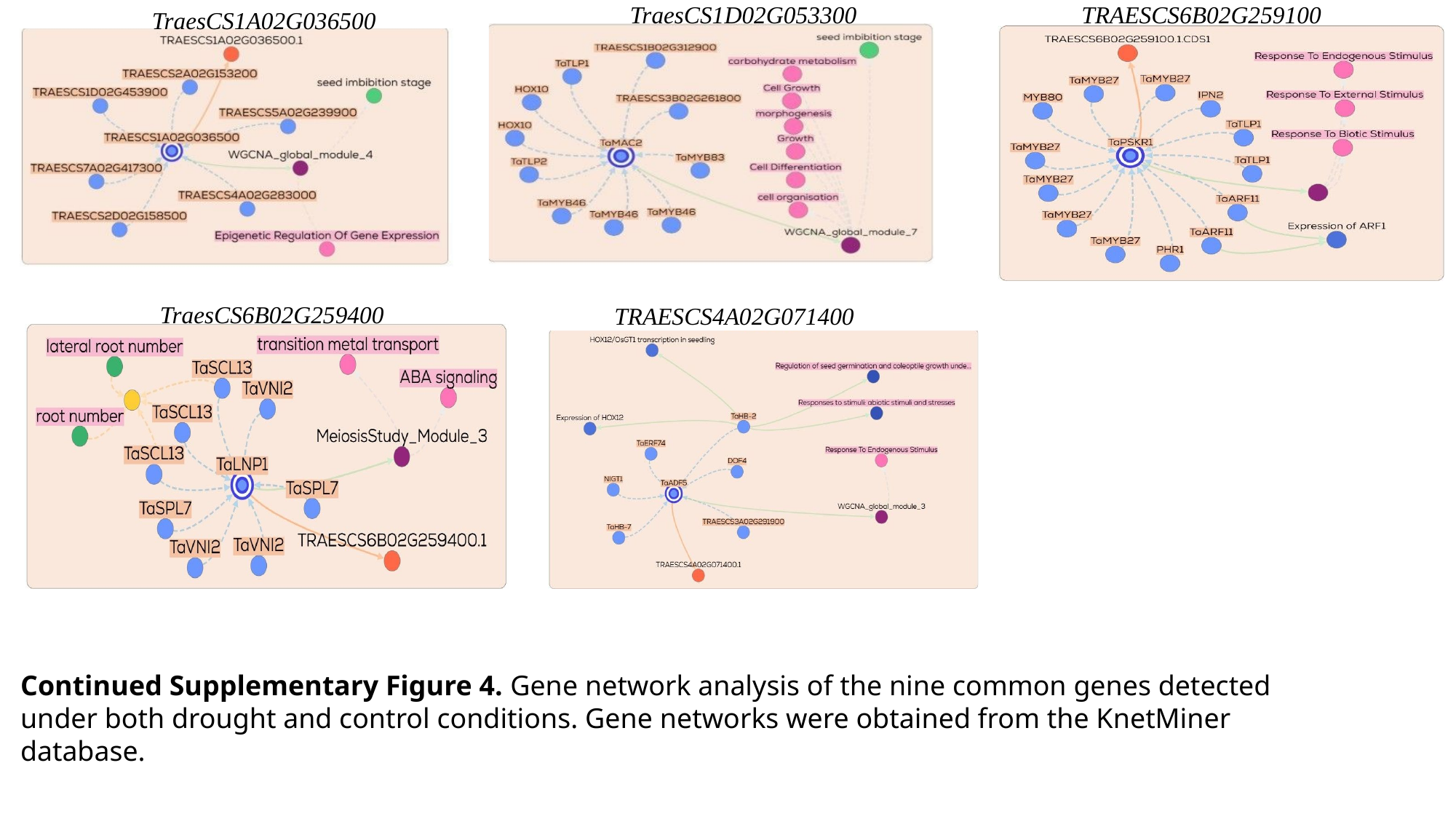

TraesCS1A02G036500
TraesCS1D02G053300
TRAESCS6B02G259100
TraesCS6B02G259400
TRAESCS4A02G071400
Continued Supplementary Figure 4. Gene network analysis of the nine common genes detected under both drought and control conditions. Gene networks were obtained from the KnetMiner database.
